# STGAT: spatial domain identification of consecutive slices based on graph contrastive learning

**DOI:** 10.1093/bib/bbag407

**Published:** 2026-07-30

**Authors:** Yuhui Feng, Shutong Xiao, Guanghua Zhou, Weiyue Ding, Boran Yang, Yiyuan Guo, Xinmo Huang, Yang Zhou, Shuilin Jin

**Affiliations:** School of Mathematics, Harbin Institute of Technology, 92 West Dazhi Street, Nangang District, Harbin, 150000, Heilongjiang, China; School of Mathematics, Harbin Institute of Technology, 92 West Dazhi Street, Nangang District, Harbin, 150000, Heilongjiang, China; School of Mathematics, Harbin Institute of Technology, 92 West Dazhi Street, Nangang District, Harbin, 150000, Heilongjiang, China; School of Mathematics, Harbin Institute of Technology, 92 West Dazhi Street, Nangang District, Harbin, 150000, Heilongjiang, China; School of Mathematics, Harbin Institute of Technology, 92 West Dazhi Street, Nangang District, Harbin, 150000, Heilongjiang, China; Department of Ophthalmology, The First Affiliated Hospital of Harbin Medical University, 23 Youzheng Street, Nangang District, Harbin 150001, Heilongjiang, China; College of Science, The University of Manchester, Oxford Road, Manchester, M13 9PL, United Kingdom; School of Mathematics, Harbin Institute of Technology, 92 West Dazhi Street, Nangang District, Harbin, 150000, Heilongjiang, China; School of Mathematics, Harbin Institute of Technology, 92 West Dazhi Street, Nangang District, Harbin, 150000, Heilongjiang, China

**Keywords:** spatial domain identification, spatial transcriptomics, canonical correlation analysis, graph contrastive learning

## Abstract

With recent advances in spatial transcriptomic technologies, multi-tissue section datasets are proliferating. While existing computational methods have achieved substantial progress in integrating multiple sections and correcting for batch effects, current approaches for spatial domain identification often fail to fully leverage both spatial context and gene expression information across consecutive sections. Moreover, prevailing graph contrastive learning frameworks typically depend on the construction of positive and negative sample pairs—a process susceptible to the introduction of noise. To overcome these limitations, we introduce STGAT, a framework that first achieves precise spatial alignment across sections using gene expression similarity. Within a unified spatial domain, STGAT employs a graph contrastive learning strategy that requires only positive pairs, enabling effective self-supervised representation learning of graph nodes. Experimental results demonstrate that STGAT effectively enhances clustering accuracy in spatial domain identification tasks across multi-section and cross-technology datasets. When applied to mouse olfactory bulb sections, the method yields sharply defined spatial domain boundaries and allows accurate identification of distinct anatomical regions. Furthermore, STGAT provides a more refined characterization of the tumor microenvironment. The source code used in this paper can be found in https://github.com/Jinsl-lab/STGAT.

## Introduction

Spatial transcriptomics (ST) [1], an emerging technology, complements traditional single-cell RNA sequencing by enabling simultaneous measurement of gene expression profiles and the spatial positions of cells within intact tissue samples, thereby preserving crucial spatial context. ST enables the simultaneous measurement of gene expression profiles and spatial positions of cells within intact tissue samples. This technology offers a novel perspective for in-depth investigation of molecular mechanisms and pathological progression in both normal and diseased states, greatly accelerating advances in biomedical research. In recent years, spatial transcriptomic technologies have been rapidly evolving. Techniques such as 10× Visium [2], Slide-seq [3], Stereo-seq [4], seqFISH+ [5], and MERFISH [6] have been introduced one after another, each demonstrating unique advantages in different application scenarios. Accurate identification of spatial domains constitutes a pivotal component of spatial transcriptomic analysis. However, precise spatial domain delineation remains challenging due to several factors, including high technical noise, data sparsity, and batch effects.

Within the algorithmic research domain, spatial domain identification techniques have progressively evolved into three major methodological frameworks. The first category comprises common clustering algorithms, including K-means [7], Leiden [8], and Louvain [9]. These algorithms can efficiently handle large-scale datasets; however, because they do not consider spatial information, they have limitations in interpreting spatially specific biological phenomena. The second category comprises algorithms based on statistical probability models. For instance, Giotto [10] employs hidden Markov random fields to model gene expression patterns. However, it has certain limitations in capturing the complex features of ST data. BayesSpace [11], on the other hand, draws on the principles of super-resolution image reconstruction in computer vision, constructing an analytical model that integrates full Bayesian statistics with Markov random fields, thereby enabling in-depth mining of spatial data. The third category of deep learning algorithms, such as stLearn [12], standardizes ST data using techniques such as neighborhood smoothing and morphological adjustment, and partitions spatial domains using graph clustering methods. However, the integration of spatial information in these methods remains suboptimal, limiting their ability to capture fine-grained tissue architecture. Some algorithms based on graph neural networks (GNNs) [13] are continually being updated: SpaceFlow [14], STAGATE [15], DeepST [16], SEDR [17], GraphST [18], SpaGCN [19]. Some graph neural network-based methods, on the other hand, employ a graph contrastive learning strategy to strengthen the feature similarity of spatially neighboring spots while suppressing associations with non-neighboring spots, thereby substantially improving the use of spatial information. However, sampling negative samples can greatly affect model performance. Recent studies have also explored graph contrastive learning in various biological contexts, such as CLSSATP [20] and MTSSMol [21], further highlighting its potential for representation learning of complex data. In ST data, spatially distant spots that exhibit similar expression may be mistakenly treated as negative samples and pushed apart, leading to suboptimal representations. Additionally, a large number of spot pair similarities must be computed, which is especially costly on large-scale graphs. Moreover, some integrated analysis tools for multiple slices have emerged, including STAligner [22], GraphST [18], and PRECAST [23]. These methods are mainly used to integrate similar or different slices and address issues such as batch effects. However, they do not fully exploit spatial and gene expression information across consecutive slices when identifying spatial domains.

To address this gap, we propose STGAT, a robust spatial-domain method for identifying consecutive slices. By constructing a dual-enhanced graph structure, it maximizes the correlation between two augmented graphs derived from the same input while simultaneously decorrelating different dimensions within each graph’s representation, thereby avoiding the traditional negative sampling process and markedly improving computational efficiency. For consecutive slice data, STGAT introduces a unified coordinate system to guide joint spatial partitioning across multiple slices. By leveraging graph structural information, self-supervised graph spot representation learning is conducted by maximizing the canonical correlation between representations of the same spot under different ‘simplified graphs’ [24]. We demonstrate STGAT’S outperformance on real datasets. Application of STGAT on the DLPFC and MERFISH dataset identified consecutive developmental. We have validated STGAT’S superior performance on across multiple datasets. Furthermore, analyzing human breast cancer sections enabled more precise identification of tumor regions.

## Material and methods

### The overview of the STGAT

**Figure 1:**
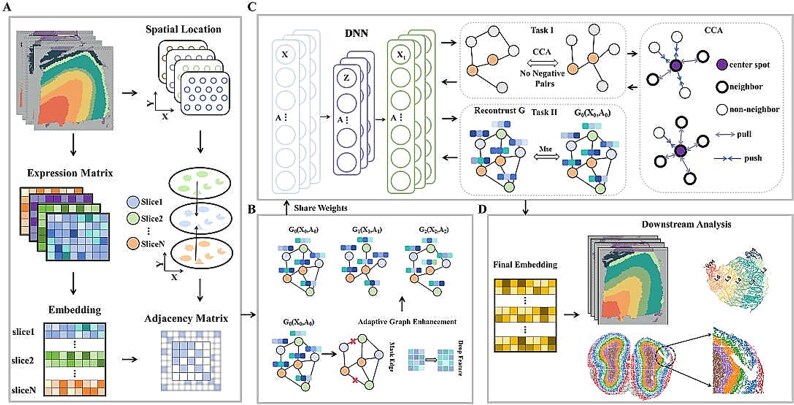
Overview of the STGAT method. (A) Using Spateo to map consecutive slices onto the same coordinate system, construct an adjacency matrix, and vertically integrate the embeddings from multiple slices. (B) Obtain the original graph and the augmented graph. (C) Input the three graphs into the DNN, enabling the model to learn low-dimensional embeddings. (D) Use the learned low-dimensional embeddings to identify the spatial domain.

We introduce STGAT (Fig. 1), a deep learning framework that integrates a graph attention network with a contrastive learning strategy to identify spatial domains across consecutive tissue sections.

This produces the original graph data. The adjacency matrix is constructed using the Delaunay triangulation [25] method. The embeddings of multiple slices are stacked vertically (Fig. 1A). Simultaneously, two augmented views are generated using random graph augmentation techniques, forcing the model to learn spatial structural invariance under these noisy conditions—that is, the same cell should maintain similar embedding representations across different augmented perspectives (Fig. 1B). The original graph and the two augmented graphs, A and B, are fed into the DNN (Deep Neural Networks); the two augmented graphs form a positive sample pair for contrastive learning, pushing the model to learn spatial structural invariance and maximize the similarity of the positive sample pair, while also constraining the independence of embedding dimensions. Finally, by weighting and fusing the contrastive learning and reconstruction losses, the model learns a low-dimensional embedding (Fig. 1C). The resulting embeddings are then used for downstream spatial domain identification (Fig. 1D). For consecutive slice data, the Spateo method is used to map consecutive slices into a common coordinate system. For single-slice data, Spateo alignment is not required; the adjacency matrix is constructed directly, and the remaining steps are the same as in the multi-slice analysis.

### Data preprocessing

We first identified highly variable genes (HVGs) using SCANPY [26]. For multi-slice datasets, the HVGs threshold was set to 9000. Given that the MERFISH dataset contained only 254 genes (fewer than 9000), all genes were retained. For single-slice datasets, 3000 HVGs were selected. Subsequently, raw data were normalized using the normalize function in the SCANPY [26] toolkit. We systematically evaluated the impact of HVG number on clustering performance. For multi-slice datasets, 9000 HVGs provided the best trade-off between retaining biological signal and computational efficiency, while 3000 HVGs sufficed for single-slice analysis (Supplementary Tables S8–S9). Finally, log-transformation was applied to the normalized expression matrix ***X***.

### Graph construction

STGAT defines the spatial neighborhood using Delaunay triangulation [25] over spot coordinates. If ***A*** is the adjacency matrix, then ***A***_***ij***_ = ***A***_***ji***_ = 1 means that spots ***i*** and ***j*** are neighbors; otherwise, ***A***_***ij***_ = ***A***_***ji***_ = 0. Self-loops are added to each spot.

Although Delaunay triangulation may occasionally produce long edges crossing tissue boundaries in sparse or damaged regions, the subsequent graph augmentation module (random edge masking) and the GATv2 self-attention mechanism can effectively down-weight such spurious connections, enhancing overall robustness. The average number of neighbors per spot for each dataset is listed in Supplementary Table S11.

We compared Delaunay triangulation with KNN and radius-based graph construction on the DLPFC dataset. Delaunay triangulation consistently achieved higher ARI and was less sensitive to parameter choices (Supplementary Table S14). Therefore, we adopted it as the default method in STGAT.

### Data augmentation

To create diverse views for contrastive learning, we generate two augmented graphs from the original graph ***G*** = (***XA***) by independently applying random masking to subsets spot features and edges. This yields two corrupted versions ***G***_***1***_ **= (*×***_***1***_**, *A***_***1***_**)** and ***G***_***2***_ **= (*×***_***2***_**, *A***_***2***_**)**, which serve as positive pairs.

### Graph attention autoencoder

The original graph data ***G*** = (***XA***) and two augmented graph ***G***_***1***_ = (***×***_***1***_, ***A***_***1***_) and ***G***_***2***_ = (***×***_***2***_, ***A***_***2***_) instances are now fed into GATE [27], which comprises an encoder-decoder architecture. The encoder generates a 64-dimensional embedding per spot, while the decoder reconstructs gene expression profiles from these embeddings. For both the encoder and decoder, the network generally employs three stacked GATv2 [28] layers. However, when processing 10× Visium [2] data—where low spatial resolution increases the risk of overfitting—we replace the architecture with a single GCN [29] layer followed by two GATv2 [28] layers. GATv2 [28] refines spot representations by considering neighboring features and their connections. Attention mechanisms weigh each neighbor’s influence, enhancing crucial spots and relationships in the updated feature set.

For a given spot ***i*** with feature vector ***h***_***i***_, we compute an attention coefficient between ***i*** and its neighboring spot ***j*** as follows: first, their features are concatenated after linear transformation, then passed through a LeakyReLU activation and a learnable weight vector ***α*** to produce a raw score:


(1) \begin{eqnarray*} {e}_{ij}=e\left({h}_i,{h}_j\right)= LeakyReLU\left({\alpha}^T\cdotp \left[W{h}_i\parallel W{h}_j\right]\right) \end{eqnarray*}

in Equation (1), ***h***_***i***_ is the input feature vector of spot ***ih***_***j***_ is the input feature ${e}_{ij}=e\left({h}_i,{h}_j\right)= LeakyReLU\left({\alpha}^T\cdotp \left[W{h}_i\parallel W{h}_j\right]\right)$vector of neighboring spot ***jα*** is the function that calculates the relevance between the two spots, and ***W*** is the weight parameter matrix for the transformation from input to output feature dimensions. In GAT, the attention mechanism is a single-layer feedforward neural network, and the activation function used is LeakyReLU. Reference [28] demonstrated that the attention mechanism in GAT is static, in which each node is only concerned with its neighbors.han In the context of dynamically selecting spots and handling structural changes comprehensively, it shows limited robustness to noise, leading to lower accuracy. Therefore, a GATv2 with a dynamic attention mechanism is proposed, and by changing the operation sequence of GAT, a general approximation relevance ***e***_***ij***_′ is obtained as shown in Equation (2).


(2) \begin{eqnarray*} {e}_{ij}^{\prime }={\alpha}^T LeakyReLU\left(\left[W{h}_i\parallel W{h}_j\right]\right) \end{eqnarray*}

To better allocate weights, softmax normalization is applied to the relevance calculated for all adjacent spots, resulting in attention coefficients ***a***_***ij***_***,*** as shown in Equation (3).


(3) \begin{eqnarray*} {\alpha}_{ij}={softmax}_i\left({e}_{ij}\right)=\frac{\mathit{\exp}\left({e}_{ij}^{\prime}\right)}{\sum_{v_k\in N\left({v}_i\right)}\mathit{\exp}\left({e}_{ik}\right)} \end{eqnarray*}

The weighted sum of ***α***_***ij***_ results in the output feature vector ***h′*** for spot ***i***, as shown in Equation (4).


(4) \begin{eqnarray*} {h}^{\prime }=\sigma \left(\sum_{v_j\in N\left({v}_i\right)}{\alpha}_{ij}W{h}_j\right) \end{eqnarray*}

Here, ***σ*** represents the activation function, typically using the $\mathrm{LeakyReLU}$ activation function. To stabilize the learning process of self-attention, GAT employs a multi-head attention mechanism to enhance model stability, performing concatenation and averaging operations, as shown in Equation (5).


(5) \begin{eqnarray*} \genfrac{}{}{0pt}{}{{\left.{h}_i^{\prime }=\right\Vert}_{m=1}^M\sigma \left(\sum_{v_j\in N\left({v}_i\right)}{\alpha}_{ij}^m{W}^m{h}_j\right)}{h_i^{\prime }=\sigma \left(\frac{1}{M}\sum_{v_j\in N\left({v}_i\right)}{\alpha}_{ij}^m{W}^m{h}_j\right)} \end{eqnarray*}

In Equation (5), ***M*** represents the number of attention heads; ‖ denotes the concatenation operation, where ***a***_***ij***_^***m***^ and ***W***^***m***^ are the attention coefficients and weight parameter matrices, respectively, for the ***m-th*** set of attention mechanisms. In the intermediate layer of GATv2, the outputs of attention heads are concatenated to capture relationships between spots and extract important features. However, in the final layer, an averaging operation is applied to reduce feature dimensions while preserving shared information across attention heads, yielding the final spot feature representation. We end up with embedding $\overset{\sim }{\boldsymbol{Z}}$, ${\overset{\sim }{\boldsymbol{Z}}}_{\mathbf{1}}$, ${\overset{\sim }{\boldsymbol{Z}}}_{\mathbf{2}}$, restructuring gene expression $\overset{\sim }{\boldsymbol{X}}$, ${\overset{\sim }{\boldsymbol{X}}}_{\mathbf{1}}$, ${\overset{\sim }{\boldsymbol{X}}}_{\mathbf{2}}$. Given a sample with ***N*** cells. Then, we normalized ${\overset{\sim }{\boldsymbol{H}}}_{\mathbf{1}}$ and ${\overset{\sim }{\boldsymbol{H}}}_{\mathbf{2}}$ by:


(6) \begin{eqnarray*} \overset{\sim }{H}=\frac{\overset{\sim }{Z}-\mu \left(\overset{\sim }{Z}\right)}{\sigma \left(\overset{\sim }{Z}\right)\sqrt{N}} \end{eqnarray*}

where ***μ*** is the mean value of each feature in the given matrix, ***σ*** indicates the s.d. of the values in each feature and ***N*** is the number of cells. Then, graph contrastive learning is as follows:


(7) \begin{eqnarray*} {L}_{con}=\parallel{\overset{\sim }{H}}_1-{\overset{\sim }{H}}_2{\parallel}_F^2+\alpha \left(\parallel{{\overset{\sim }{H}}_1}^{\top }{\overset{\sim }{H}}_1-I{\parallel}_F^2+\parallel{{\overset{\sim }{H}}_2}^{\top }{\overset{\sim }{H}}_1-I{\parallel}_F^2\right) \end{eqnarray*}

The first term encourages the embeddings from the two augmented views to be consistent, while the second term (with hyperparameter ***α***) forces the feature dimensions within each view to be decorrelated, reducing redundancy. This avoids the need for negative sampling. The reconstruction loss is as follows:


(8) \begin{eqnarray*} {L}_{rec}=\sum_{i=1}^N\parallel{X}_i-\overset{\sim }{X_i}{\parallel}^2 \end{eqnarray*}

### Model optimization

The training process jointly optimizes ***L***_***con***_ and ***L***_***rec***_ by assigning different weights to the loss function components. The final training objective of the model in this paper is defined as:


(9) \begin{eqnarray*} L={\beta}_1{L}_{con}+{\beta}_2{L}_{rec} \end{eqnarray*}

Where ***β***_***1***_ and ***β***_***2***_ are the weights of different loss functions. We use the final embeddings $\overset{\sim }{\boldsymbol{Z}}$ to perform spatial clustering. Spatial domains are identified based on the final embeddings $\overset{\sim }{\boldsymbol{Z}}$ using K-means. We performed a grid search on β₁ and β₂ across multiple datasets and found that setting both to 1.0 achieved near-optimal performance in most cases, suggesting that the contrastive learning and reconstruction tasks are of comparable importance. Ablation studies on the DLPFC dataset confirmed that deviating from this balance degrades clustering accuracy (Supplementary Table S6). The complete parameter configurations for all datasets are provided in Supplementary Table S5.”

### Baselines and evaluation metrics

The most popular spatial domain recognition algorithm was selected as the baseline. For consecutive slices data, we selected STAligner, STAGATE, and SEDR. For single slice data, we selected STAGATE, DeepST, SEDR, GraphST, SpaGCN, and Scanpy. For the sake of fairness, all parameter values are set to take the best values suggested by the original studies.

The spatial domain was assessed by two common clustering metrics. We used the Adjusted Rand Index (ARI) and Normalized Mutual Information (NMI) [30] for datasets with manually annotated labels and the Averaged Silhouette Coefficient (SC) [31] and the Davies–Bouldin Index (DB) [32] for datasets with only coarse labels. ARI is a metric that measures the similarity between ground truth labels and the clustering algorithm‘s results. The formula is as follows:


(10) \begin{eqnarray*} ARI=\frac{\sum_{ij}\kern0.1em \left(\genfrac{}{}{0pt}{}{n_{ij}}{2}\right)-\left[\sum_i\kern0.1em \left(\genfrac{}{}{0pt}{}{a_i}{2}\right)\sum_j\kern0.1em \left(\genfrac{}{}{0pt}{}{b_j}{2}\right)\right]/\left(\genfrac{}{}{0pt}{}{n}{2}\right)}{\frac{1}{2}\left[\sum_i\kern0.1em \left(\genfrac{}{}{0pt}{}{a_i}{2}\right)+\sum_j\kern0.1em \left(\genfrac{}{}{0pt}{}{b_j}{2}\right)\right]-\left[\sum_i\kern0.1em \left(\genfrac{}{}{0pt}{}{a_i}{2}\right)\sum_j\kern0.1em \left(\genfrac{}{}{0pt}{}{b_j}{2}\right)\right]/\left(\genfrac{}{}{0pt}{}{n}{2}\right)} \end{eqnarray*}

where ***n***_***ij***_ represents the number of sample pairs that fall into the same cluster in both the true labels and the clustering results.

NMI is an important metric in machine learning for evaluating the consistency between clustering results and true labels.


(11) \begin{eqnarray*} NMI\left(X,Y\right)=\frac{I\left(X,Y\right)}{\sqrt{H(X)H(Y)}} \end{eqnarray*}

where ***I(X,Y)*** denotes the mutual information between ***X*** and ***Y***, and ***H(X)*** and ***H(Y)*** represent the entropies of ***X*** and ***Y***, respectively.

The SC value ranges from −1 to 1, with a higher value indicating that the sample is better matched to its own cluster and less matched to neighboring clusters. The averaged SC for all samples can be used to evaluate clustering performance. The averaged SC is represented as follows:


(12) \begin{eqnarray*} SC=\frac{b_i-{a}_i}{\max \left({a}_i,{b}_i\right)},i\in sample\ \left\{1,2,\dots N\right\} \end{eqnarray*}

where ***a***_***i***_ denotes the average distance from sample ***i*** to other samples in the cluster, which reflects the dissimilarity within the cluster, and ***b***_***i***_ denotes the distance from sample ***i*** to the nearest outer cluster.

### Identification and functional analysis of differentially expressed genes

Differentially expressed genes (DEGs) between spatial domains were identified using the Wilcoxon rank-sum test implemented in Scanpy. Raw *P*-values were corrected for multiple testing with the Benjamini–Hochberg (BH) procedure to control the false discovery rate. For the human breast cancer dataset, where we aimed to capture subtle expression variations within the tumor microenvironment, genes with |log₂ fold change| > 0.25 and BH-adjusted *P* < .05 were considered significant. For all other datasets, a more stringent threshold of |log₂ fold change| > 2 and BH-adjusted *P* < .05 was applied to highlight robustly DEGs.

## Results

### STGAT identifies the spatial domain on consecutive slices

**Figure 2:**
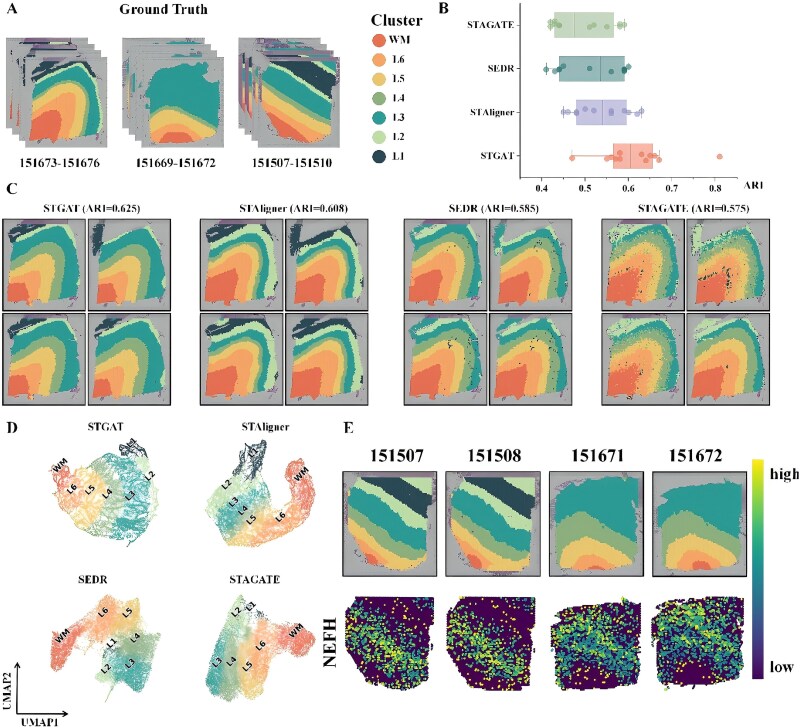
Spatial domain identification on the DLPFC dataset, UMAP visualization. (A) Manual annotation of 12 slices from the DLPFC dataset. (B) Box plots of ARI for the 12 slices from the DLPFC dataset identified by four methods. (C) Visualization of spatial domains identified by STGAT, STAligner, SEDR, and STAGATE in slices 151 673–151 676. (D) UMAP visualizations of the four methods. (E) Visualization of spatial domains in slices 151 507–151 508 and 151 671–151 672 identified by STGAT, and the expression of the NEFH gene in the third layer.

To evaluate STGAT’s performance in identifying spatial domains across consecutive sections, we used the LIBD human dorsolateral prefrontal cortex (DLPFC) dataset, which comprises spatial transcriptomic profiles from 12 DLPFC slices. The ground truth annotations for a representative slice are shown in Fig. 2A. We assessed clustering accuracy using the ARI.

Moreover, we selected advanced algorithms capable of multi-slice analysis for comparison, namely STAligner, STAGATE and SEDR. The spatial domain identification results were evaluated across four consecutive sections, comparing all four methods. Notably, only STGAT and STAligner were able to clearly identify seven layers, whereas STAGATE and SEDR could only identify six layers (Fig. 2C). Furthermore, STGAT achieved the highest ARI = 0.625. Results for the remaining slices can be found in Supplementary Fig. 1.

As summarized in the box plot in Fig. 2B, which compares the ARI values of spatial domain identification across the four methods, STGAT achieved the highest median ARI. Detailed ARI metrics for each individual slice are available in Supplementary Table S1.

STGAT effectively enhances feature discriminability by maximizing mutual information between graphs while minimizing redundancy within each graph. UMAP [33] visualization of the enhanced features accurately captured spatial domain relationships and revealed clear trajectories. As shown in Fig. 2D, the UMAP embedding for slices 151,673–151,676 not only faithfully reconstructed the relative positions of spots and spatial domains but also exhibited well-separated boundaries between different domains. We then used the trajectory inference algorithm PAGA [34] to validate the inferred trajectories (Supplementary Fig. 2). The PAGA graphs generated by STGAT and STAligner clearly depict a near-linear developmental trajectory from layer 1 to layer 6 and WM, while also reflecting similarities between adjacent layers. In contrast, the PAGA results from by STAGATE and SEDR show some mixing across layers.

To validate the clustering results, we compared the distribution of marker genes with the spatial domains identified by STGAT (Fig. 2E). We selected four slices: 151 507–151 508 and 151 671–151 672, with results for the remaining slices shown in Supplementary Fig. 3. The results demonstrated that most aggregation regions closely matched the expression patterns of their corresponding marker genes. Specifically, based on the expression characteristics of the NEFH gene [35], STGAT accurately reconstructed the spatial domain of the third-layer region; this gene is closely related to the distribution characteristics of neural structures, with similar results observed in the other slices. These results demonstrate that STGAT shows considerable advantages in spatial domain identification in the DLPFC consecutive slice dataset, effectively validating its superior spatial domain clustering capability and biological structure analysis performance.

Beyond clustering accuracy, we evaluated the computational efficiency of STGAT on the DLPFC dataset (slices 151 673–151 676). Compared to STAGATE, STGAT achieved higher ARI while reducing GPU memory consumption by ~40% (6.26 GB versus 10.48 GB). Although SEDR was faster (10 s), its clustering accuracy was substantially lower. STAligner required less training time (1.35 min) but used slightly more memory (6.89 GB). These results demonstrate STGAT’s favorable trade-off among accuracy, time, and memory (Supplementary Table S12).

### STGAT enhanced analysis of consecutive slices in MERFISH data

**Figure 3:**
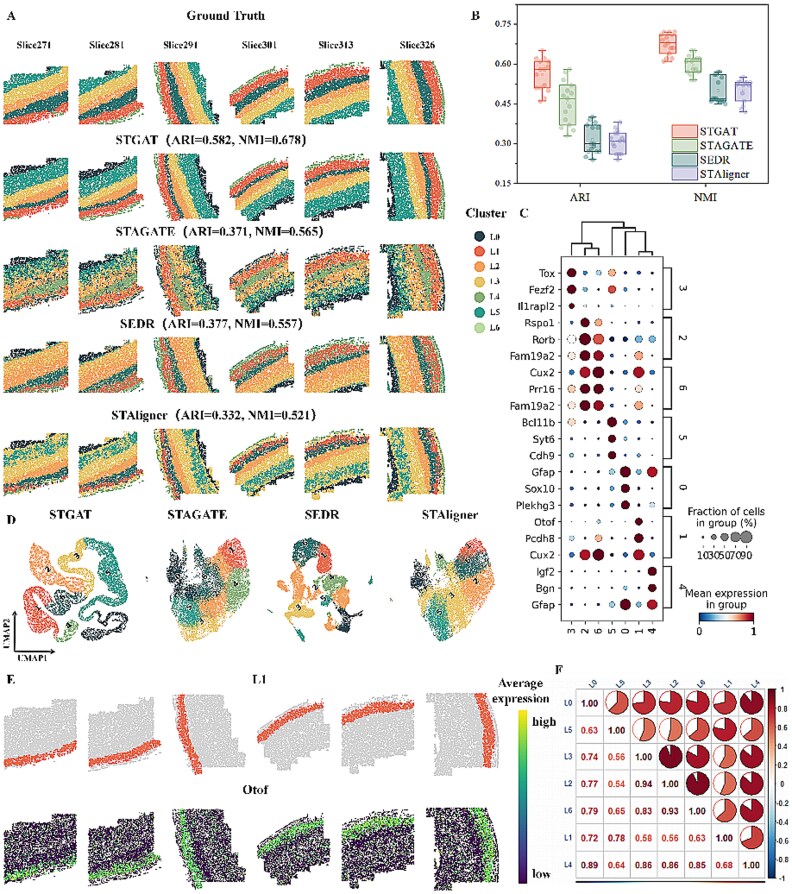
STGAT demonstrates strong performance on the MERFISH dataset. (A) Manual annotations and spatial domain identification results of six slices from sample 6 in the MERFISH dataset using four methods. (B) Box plots of the identification results of the 15 slices from the MERFISH dataset by the four methods. (C) Highly expressed DEGs in each layer. (D) UMAP visualizations of the four methods. (E) Visualization of spatial domains identified by STGAT in the six slices of sample 6 and L1 expression of the Otof gene. (F) Heatmap of Pearson correlation coefficients between domains.

We next evaluated STGAT’S performance in identifying spatial domains using a MERFISH dataset from SPACEL. Our analysis included a total of fifteen slices from samples 2, samples 3, and samples 6. Comparative results across all slices demonstrated that STGAT outperformed other methods in analyzing the MERFISH dataset (Fig. 3B). Representative results from sample 6 are visualized in Fig. 3A, which shows that STGAT produced sharply defined spatial domain boundaries, whereas those identified by STAGATE, SEDR, and STAligner appeared blurred. Consistent findings were observed across additional tissue sections (Supplementary Figs 4–5) UMAP visualizations (Fig. 3D and Supplementary Fig. 6) further support these conclusions. Additionally, clustering performance was assessed using Silhouette Coefficient (SC) and Davies–Bouldin Index (DB), with STGAT achieving the best scores (Supplementary Fig. 8). These results demonstrate the robust performance of STGAT in spatial domain identification.

DEGs with high expression in each layer were filtered using the Wilcoxon signed-rank test. Otof was highly expressed in layer 1 (Fig. 3C and Supplementary Fig. 7), consistent with previous studies [36]. We also mapped the distribution of marker genes and compared it with the spatial domains identified by STGAT, finding a high degree of concordance (Fig. 3E). Finally, we calculated the Pearson correlation coefficients between domains and found that there was a strong correlation within each domain (Fig. 3F and Supplementary Fig. 9), STGAT successfully captures the biological continuity and gradients within the tissue.

### STGAT enhanced analysis of consecutive slices in MERFISH data

In this subsection, we evaluate the performance of the STGAT method on mouse olfactory bulb (MOB) datasets generated by Stereo-seq and Slide-seqV2. For benchmarking, six additional single-slice spatial clustering methods were selected, including five spatial clustering methods—SEDR, GraphST, DeepST, SpaGCN, and STAGATE—as well as one non-spatial clustering method from SCANPY.

**Figure 4:**
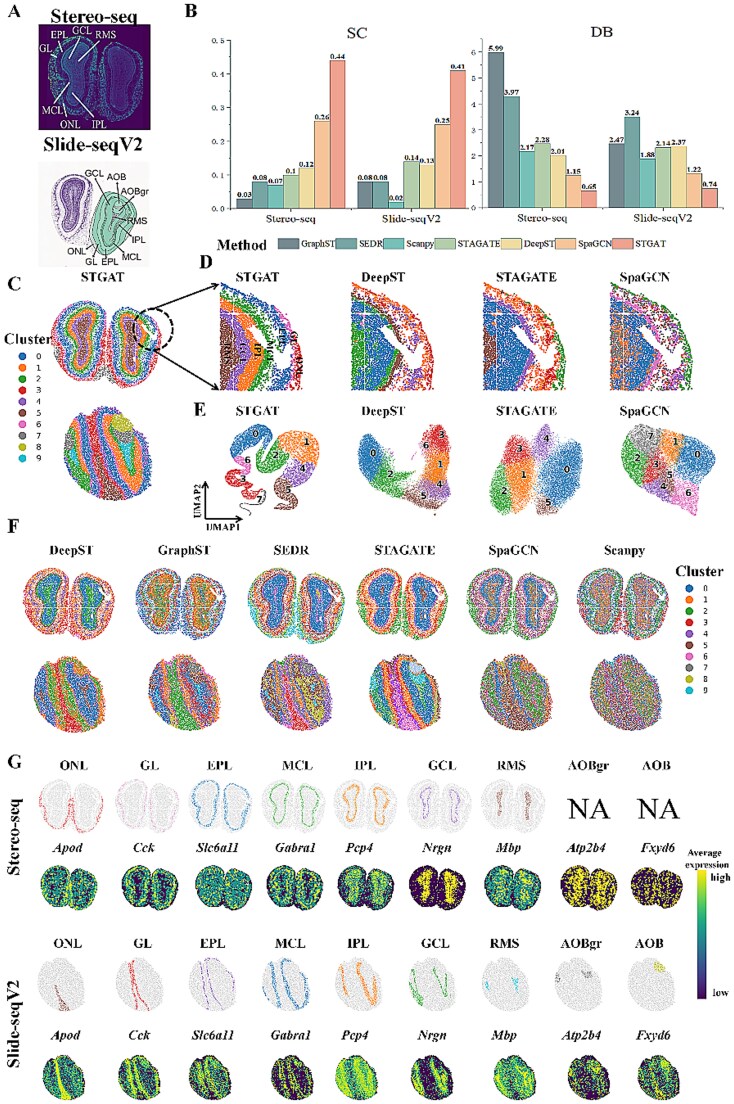
STGAT is applicable to ST datasets generated from different platforms. (A) An annotated map of the laminar organization of the mouse olfactory bulb on DAPI-stained images and images of the mouse olfactory bulb from the Allen Mouse Brain Atlas. (B) Comparison of clustering metrics for different methods. (C) Spatial domain identification by STGAT on both platforms. (D) The magnified region of interest shows the results for low-quality spots processed by different methods. (E) UMAP results by four methods: STGAT, DeepST, STAGATE, and SpaGCN. (F) Results of spatial domain identification by six methods. (G) STGAT identified spatial domains and corresponding marker genes in slices from both platforms (Stereo-seq and Slide-seqV2).

The Stereo-seq platform utilizes DNB chips and in situ RNA capture to enable spatial omics profiling with both wide-field and nanoscale resolution [4]. The layered architecture of the coronal mouse olfactory bulb, as revealed by DAPI staining (top of Fig. 4A). Due to the absence of spot-level fine-grained annotation, spatial domain identification performance was assessed using widely adopted unsupervised evaluation metrics SC and DB. STGAT achieved optimal scores for both metrics (SC = 0.44 and DB = 0.65), outperforming all benchmarked methods (Fig. 4B). Considering that SC and DB alone are insufficient to evaluate spatial relationships between domains, we further compared the spatial domains and expression distributions of previously known layer marker genes shown above in Fig. 4G [37]. STGAT was the only method that cleanly separated the clustering boundaries of each spatial domain (top of Fig. 4C). UMAP visualizations for STGAT, DeepST, STAGATE, and SpaGCN are presented in Fig. 4E, with additional results provided in Supplementary Fig. 10. The embeddings generated by STGAT revealed progressive and consecutive transitions between MOB regions—a pattern not observed in the representations from other methods. These results underscore STGAT’s strong competitive advantage in identifying spatially coherent and biologically meaningful domains.

We further applied STGAT to a Slide-seqV2 mouse olfactory bulb dataset (Fig. 4A, bottom), which offers a higher spatial resolution of 10 μm. In addition to the spatial areas contained in the Stereo-seq platform data, the dataset generated by Slide-seqV2 also includes two special spatial structures: accessory olfactory bulb (AOB) and accessory olfactory bulb granule layer (AOBgr) [18]. STGAT achieved optimal clustering performance, as reflected by the best scores for both silhouette coefficient (SC = 0.41) and Davies-Bouldin index (DB = 0.74) among all methods (Fig. 4B). Below Fig. 4C and Fig. 4F show the results of spatial domain identification by seven different methods. Notably, only STGAT and STAGATE successfully identified the AOB and AOBgr domains. However, the boundaries delineated by STAGATE were noticeably blurred compared to the sharply defined domains produced by STGAT. Consistently, the spatial domains identified by STGAT showed strong agreement with the expression patterns of established layer-specific marker genes (Fig. 4G, bottom). Specifically, the AOB and AOBgr domains were validated by the distinct expression of Fxyd6 and Atp2b4, respectively, further confirming the biological accuracy of STGAT.

Overall, the test results indicate that STGAT enhances the model’s generalization ability and improves its capability to recognize spatial domains with different spatial resolutions in ST data. Notably, despite the substantial resolution difference between Stereo-seq and Slide-seqV2 (10 μm), STGAT with Delaunay triangulation achieved top performance on both platforms, underscoring the robustness of its graph construction strategy to varying spatial sampling densities.

### Performance of STGAT in identifying the spatial domain of the breast cancer ST dataset

**Figure 5:**
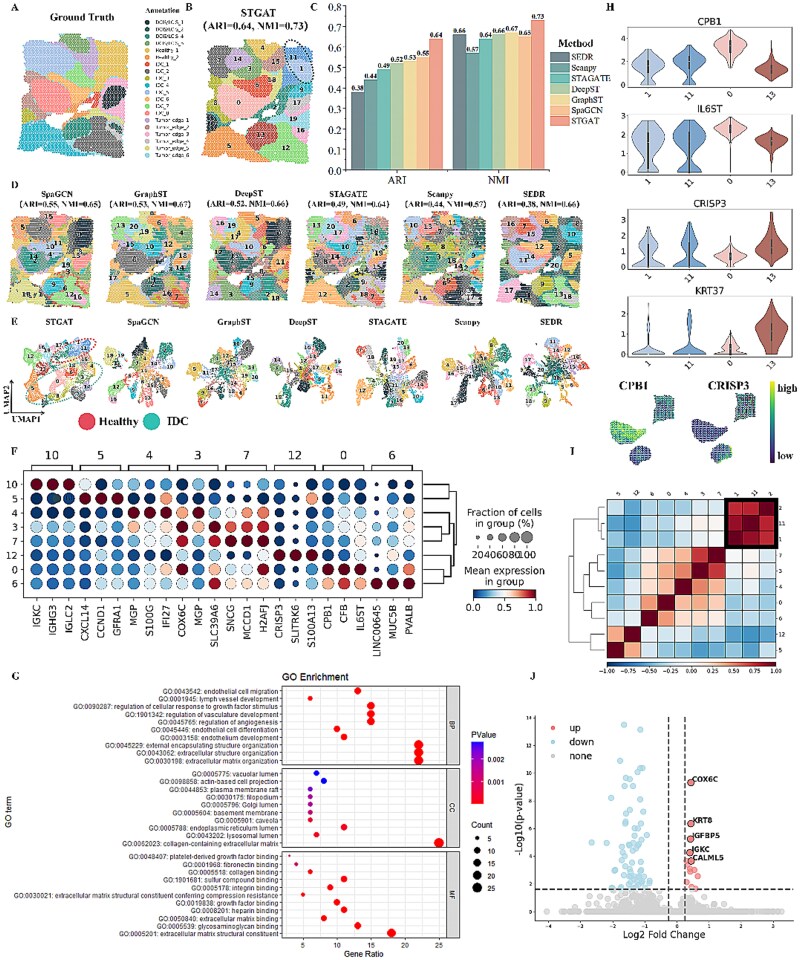
Performance of STGAT in identifying spatial domains in the breast cancer ST dataset. (A) Manual annotation of a human breast cancer sample. (B) Spatial domain identification results of the breast cancer sample by STGAT. (C) Comparison of clustering metrics by different methods. (D) Spatial domain identification results by six methods. (E) Result of UMAP by the seven methods. (F) DEG heatmap for the IDC domain. (G) GOEA of DEGs between domains 18 and 15. (H) Differential expression of CPB1, IL6ST, CRISP3, and KRT37 between domains 1, 11, 0, and 13 (top); visualization of CPB1 and CRISP3 between domains 1, 11, 0, and 13. (I) Heatmap of Pearson correlation coefficient between healthy regions and IDC. (J) Volcano plot of DEGs between domains 1 and 11.

Given the complex microarchitecture of cancerous tissues, we evaluated STGAT alongside six other methods using publicly available ST data from breast invasive ductal carcinoma (IDC), to assess their ability to resolve intratumoral structures. The tissue was histologically categorized into four main regions (Fig. 5A): ductal carcinoma in situ (DCIS), healthy tissue, invasive ductal carcinoma (IDC), and the tumor margin. The spatial domain identification results from STGAT and the other methods are shown in Fig. 5B and D. STGAT exhibited the closest agreement with manual histological annotations and more clearly delineated distinct tumor regions compared to other approaches. As illustrated in Fig. 5C, STGAT also achieved the highest scores in both ARI (0.64) and NMI (0.73). Furthermore, UMAP visualization of STGAT embeddings clearly separated IDC regions from healthy tissue (Fig. 5E). Additionally, we compared five other methods—stHGC [38], MVST [39], ST-GCP [40], MMSpa [41] and TOGAR [42]—and the spatial domains identified by these methods are presented in Supplementary Fig. 16.

Notably, STGAT proposes a slightly different but more refined spatial stratification. Specifically, STGAT subdivided the manually annotated region ‘Healthy_1’ (Fig. 5A) into two distinct sub-domains (labeled 1 and 11 in Fig. 5B). Comparative analysis of these sub-domains (Fig. 5G and J) revealed significantly elevated expression of KRT8, IGFBP5, and CALML5 in domain 1 relative to domain 11. IGFBP5, a member of the insulin-like growth factor binding protein family, is known to regulate IGF signaling pathways. KRT8, an intermediate filament protein critical to epithelial cytoskeletal integrity, is involved in the assembly of extracellular structures such as the basement membrane.

To quantify intratumoral heterogeneity, we computed pearson correlation coefficients between transcriptional profiles. This analysis revealed substantial heterogeneity, with domains 1, 2, and 11 exhibiting distinct expression patterns compared to all other domains (Fig. 5I). Here, we mainly compared domain 13 (ductal/lobular carcinoma, DCIS/LCIS), domain 0 (invasive ductal carcinoma, IDC), and domains 1 and 11 (healthy regions) through differential expression and pathway enrichment analysis (Fig. 5H and Supplementary Figs 13–15), This integrated analysis identified key marker genes (CRISP3, IL6ST, KRT37, CPB1) whose expression patterns strongly aligned with and accurately delineated their respective pathological regions (Fig. 5H).

To comprehensively understand the biomarker genes in the spatial domain, we performed differential expression analysis to obtain DEGs. Figure 5F shows the top 3 DEGs of IDC. This section confirms that applying STGAT to analyze cancer ST datasets can reveal local gene expression variations in the tumor microenvironment, providing new insights into the mechanisms of cancer spatial heterogeneity.

## Discussions

This study proposes a spatial domain recognition method for multi-task learning in graph contrastive learning, based on consecutive slicing and integrated with canonical correlation analysis—STGAT. Furthermore, we evaluated the robustness of STGAT against potential alignment errors introduced by the external tool Spateo. By artificially perturbing the aligned coordinates of DLPFC slices with Gaussian noise, we observed that STGAT maintained high clustering performance (ARI remained above 0.5 even under moderate noise), demonstrating its resilience to initial alignment deviations (Supplementary Tables S3–S4).

To evaluate the performance of STGAT, we conducted experiments on datasets from four platforms: 10× Visium, Stereo-seq, Slide-seqV2, and MERFISH. STGAT can effectively integrate spatial and gene expression information from consecutive slices by constructing dual-augmented views and maximizing the canonical correlation between positive sample pairs, thereby avoiding the noise issues caused by negative sampling in traditional contrastive learning. Experimental results show that STGAT outperforms current state-of-the-art methods on both single-slice and multi-slice data from multiple platforms (including 10× Visium, Slide-seq, Stereo-seq, and MERFISH), demonstrating excellent clustering accuracy, generalization capability, and biological interpretability. STGAT not only provides a novel technical solution but also offers a powerful tool for understanding tissue architecture and disease microenvironments.

Firstly, the multi-slice alignment strategy implemented in Spateo considerably enhances cross-sample consistency, particularly suitable for biologically consecutive tissues such as the cerebral cortex. Secondly, the contrastive learning strategy using only positive sample pairs not only reduces computational burden but also avoids biases introduced by improper negative sampling—this is especially important in ST, where spatially distant spots may still exhibit similar gene expression patterns. STGAT can not only accurately identify spatial domains but also highly align with the expression patterns of known marker genes, demonstrating strong analytical capabilities, particularly in complex tissues like tumor microenvironments. For example, in breast cancer data, STGAT successfully revealed heterogeneity between healthy and tumor tissues and identified gene modules associated with disease progression.

Although STGAT performs excellently in many aspects, it still has some limitations. For instance, the current multi-slice alignment still relies on external tools (Spateo), and future work could explore an end-to-end framework that jointly optimizes alignment and representation learning. Additionally, computational efficiency for extremely large-scale datasets still requires further optimization. We plan to explore data-adaptive graph construction methods, such as dynamically adjusting distance thresholds or applying pruning strategies based on local spot density, to further enhance boundary delineation in regions with extreme non-uniform sampling.

**Key Points**

- STGAT employs a graph contrastive learning strategy based on canonical correlation analysis, utilizing only positive sample pairs to avoid noise from negative sampling, and effectively integrates spatial and gene expression information across consecutive sections.
- By constructing dual-augmented views, maximizing their canonical correlation, and decorrelating representation dimensions, STGAT learns robust and discriminative low-dimensional embeddings of spatial tissue architecture.
- STGAT demonstrates superior performance in spatial domain identification over existing state-of-the-art methods on multi-platform datasets (10× Visium, Slide-seq, Stereo-seq, and MERFISH).
- The method exhibits exceptional fine-grained resolution in complex tissues, such as the tumor microenvironment, enabling the identification of biologically meaningful subregions that align with marker gene expression.

## Supplementary Material

Supplementary_Materials_bbag407

## Data Availability

We validate the performance of our proposed STGAT using SRT datasets from four platforms, which are available on the public website. **● Human dorsolateral pre-frontal cortex (DLPFC):** The 10× Visium dataset comprises 12 slices, and all slices were manually annotated. The data links to http://spatial.libd.org/spatialLIBD. **● Human breast cancer:** The 10× Visium dataset comprises 3798 spots and 36,601 genes, with 20 regions manually annotated. The data links to https://www.10xgenomics.com/cn/datasets/human-breast-cancer-block-a-section-1-1-standard-1-1-0. **● Mouse olfactory bulb:** The mouse olfactory bulb tissue data generated by Stereo- seq and Slide-seqV2 platforms can be accessed from https://github.com/JinmiaoChenLab/SEDR_analyses and https://singlecell.broadinstitute.org/single_cell/study/SCP815, respectively. **● Mouse Brain:** The MERFISH data comprises 15 slices, with 254 genes and 5–7 regions manually annotated. The data links to https://doi.brainimagelibrary.org/doi/10.35077/g.21.
