# Supplementary material for: STGAT: spatial domain identification of consecutive slices based on graph contrastive learning
Source: Brief Bioinform. 2026 Jul 30;27(4):bbag407. doi: 10.1093/bib/bbag407 (PMC13435228; doi:10.1093/bib/bbag407)
Supplement: Supplementary file 1 [file supplementary_materials_bbag407.docx]

**Supplementary Materials**

Yuhui Feng^1, #^, Shutong Xiao^1, #^, Guanghua Zhou^1, #^, Weiyue Ding^1, #^, Boran Yang^1^, Yiyuan Guo^2^, Xinmo Huang^3^, Yang Zhou^1, *^, Shuilin Jin^1, *^

^1^School of Mathematics, Harbin Institute of Technology, Harbin 150000, China

^2^Department of Ophthalmology, The First Affiliated Hospital of Harbin Medical University, Harbin, China 150001, China

^3^College of Science, The University of Manchester, Oxford Road, Manchester, M13 9PL, UK

^*^To whom correspondence should be addressed. E-mail(s): [yangz@hit.edu.cn](mailto:yangz@hit.edu.cn), jinsl@hit.edu.cn

^#^The authors wish it to be known that, in their opinion, the first four authors should be regarded as Joint First Authors.

**Supplementary Figures**

**Supplementary Figure 1.** Ground Truth and comparison of spatial domains by clustering assignments via STGAT, STAligner, STAGATE and SEDR in Slice 151507-151510 and 151669-151672 of the DLPFC dataset.

**Supplementary Figure 2.** PAGA trajectory inference results generated from the embeddings of STGAT, STAligner, SEDR and STAGATE on slice 151673-151676 of the DLPFC dataset.

**Supplementary Figure 3.** Visualization of the spatial domains identified by STGAT on slice 151509-151510, 151669-151670 and 151673-151676 of the DLPFC dataset and the corresponding marker gene expressions.

**Supplementary Figure 4.** Ground Truth and comparison of spatial domains by clustering assignments via STGAT, STAligner, STAGATE and SEDR in Slice 62, 71, 81, 91 and 102 of the Merfish dataset.

**Supplementary Figure 5.** Ground Truth and comparison of spatial domains by clustering assignments via STGAT, STAligner, STAGATE and SEDR in Slice 112, 121, 131 and 153 of the Merfish dataset.

**Supplementary Figure 6.** UMAP visualization of latent features for Sample2 and Sample3 of the Merfish dataset by four methods.

**Supplementary Figure 7.** sample2 and sample3 were highly expressed DEGs in each layer.

**Supplementary Figure 8.** Barchart of SC and DB values across sample2, sample3 and sample6 of the Merfish dataset for 4 methods.

**Supplementary Figure 9.** Heat map of Pearson correlation coefficients between domains.

**Supplementary Figure 10.** GraphST, SEDR and Scanpy three methods UMAP visualization and corresponding part display.

**Supplementary Figure 11.** Seven methods UMAP visualization.

**Supplementary Figure 12.** STGAT identifies the expression distribution of biological genes in each spatial domain.

**Supplementary Figure 13.** GO enrichment analysis and volcanic map of DEGS between domains 0 and 13.

**Supplementary Figure 14.** GO enrichment analysis and volcanic map of DEGS between domains 1, 11 and 0.

**Supplementary Figure 15.** GO enrichment analysis and volcanic map of DEGS between domains 1, 11 and 13.

**Supplementary Figure 16.** Spatial domain identification results by eight methods.

**Supplementary Tables**

**Supplementary Table 1.** STGAT, STAligner, SEDR and STAGATE identify the ARI values of the spatial domain for twelve slices

|  | **STGAT** | **STAligner** | **SEDR** | **STAGATE** |
| --- | --- | --- | --- | --- |
| **151507** | **0.55** | 0.48 | 0.41 | 0.42 |
| **151508** | **0.56** | 0.48 | 0.43 | 0.44 |
| **151509** | 0.47 | 0.52 | **0.59** | 0.51 |
| **151510** | 0.58 | 0.50 | **0.59** | 0.52 |
| **151669** | **0.67** | 0.45 | 0.45 | 0.43 |
| **151670** | **0.65** | 0.46 | 0.44 | 0.44 |
| **151671** | **0.81** | 0.56 | 0.44 | 0.43 |
| **151672** | **0.58** | 0.56 | 0.51 | 0.42 |
| **151673** | **0.66** | 0.63 | 0.60 | 0.59 |
| **151674** | **0.63** | 0.59 | 0.59 | 0.58 |
| **151675** | 0.57 | **0.62** | 0.59 | 0.58 |
| **151676** | **0.64** | 0.60 | 0.56 | 0.55 |

**Supplementary Table 2.** The statistics of the datasets.

| **Datasets** | **Organisms** | **Slices** | **Spots** | **Genes** | **Domains** | **Platforms** |
| --- | --- | --- | --- | --- | --- | --- |
| **DLPFC_151507** | Human | 1 | 4226 | 33538 | 7 | 10X Visium |
| **DLPFC_151508** | Human | 1 | 4384 | 33538 | 7 | 10X Visium |
| **DLPFC_151509** | Human | 1 | 4789 | 33538 | 7 | 10X Visium |
| **DLPFC_151510** | Human | 1 | 4634 | 33538 | 7 | 10X Visium |
| **DLPFC_151669** | Human | 1 | 3661 | 33538 | 5 | 10X Visium |
| **DLPFC_151670** | Human | 1 | 3498 | 33538 | 5 | 10X Visium |
| **DLPFC_151671** | Human | 1 | 4110 | 33538 | 5 | 10X Visium |
| **DLPFC_151672** | Human | 1 | 4015 | 33538 | 5 | 10X Visium |
| **DLPFC_151673** | Human | 1 | 3639 | 33538 | 7 | 10X Visium |
| **DLPFC_151674** | Human | 1 | 3673 | 33538 | 7 | 10X Visium |
| **DLPFC_151675** | Human | 1 | 3592 | 33538 | 7 | 10X Visium |
| **DLPFC_151676** | Human | 1 | 3460 | 33538 | 7 | 10X Visium |
| **Sample2_slice62** | Mouse brain | 1 | 4792 | 254 | 6 | MERFISH |
| **Sample2_slice71** | Mouse brain | 1 | 5749 | 254 | 6 | MERFISH |
| **Sample2_slice81** | Mouse brain | 1 | 5262 | 254 | 6 | MERFISH |
| **Sample2_slice91** | Mouse brain | 1 | 5661 | 254 | 6 | MERFISH |
| **Sample2_slice102** | Mouse brain | 1 | 6731 | 254 | 6 | MERFISH |
| **Sample3_slice112** | Mouse brain | 1 | 4996 | 254 | 7 | MERFISH |
| **Sample3_slice122** | Mouse brain | 1 | 6185 | 254 | 7 | MERFISH |
| **Sample3_slice131** | Mouse brain | 1 | 5061 | 254 | 7 | MERFISH |
| **Sample3_slice153** | Mouse brain | 1 | 7626 | 254 | 7 | MERFISH |
| **Sample6_slice271** | Mouse brain | 1 | 3899 | 254 | 7 | MERFISH |
| **Sample6_slice283** | Mouse brain | 1 | 4308 | 254 | 7 | MERFISH |
| **Sample6_slice291** | Mouse brain | 1 | 4453 | 254 | 7 | MERFISH |
| **Sample6_slice301** | Mouse brain | 1 | 4408 | 254 | 7 | MERFISH |
| **Sample6_slice313** | Mouse brain | 1 | 3920 | 254 | 7 | MERFISH |
| **Sample6_slice326** | Mouse brain | 1 | 4145 | 254 | 7 | MERFISH |
| **Olfactory bulb** | Mouse | 1 | 19109 | 14376 | 7 | Stereo-seq |
| **Olfactory bulb** | Mouse | 1 | 20139 | 18675 | 9 | Slide-seqV2 |
| **Breast cancer** | Human | 1 | 3798 | 36601 | 20 | 10X Visium |

**Supplementary Table S3.** ARI values under different noise levels

| **K** | **ARI Mean** | **151673** | **151674** | **151675** | **151676** |
| --- | --- | --- | --- | --- | --- |
| **0** | **0.63** | **0.66** | **0.63** | **0.57** | **0.64** |
| **0.1** | 0.58 | 0.58 | 0.60 | 0.57 | 0.56 |
| **0.2** | 0.48 | 0.47 | 0.52 | 0.48 | 0.46 |
| **0.3** | 0.57 | 0.54 | 0.59 | 0.58 | 0.57 |
| **0.4** | 0.53 | 0.53 | 0.55 | 0.51 | 0.55 |
| **0.5** | 0.54 | 0.52 | 0.56 | 0.53 | 0.57 |

**Supplementary Table S4.** NMI values under different noise levels

| **K** | **NMI Mean** | **151673** | **151674** | **151675** | **151676** |
| --- | --- | --- | --- | --- | --- |
| **0** | **0.71** | **0.72** | **0.72** | **0.69** | **0.72** |
| **0.1** | 0.70 | 0.70 | 0.71 | 0.70 | 0.69 |
| **0.2** | 0.61 | 0.60 | 0.63 | 0.61 | 0.61 |
| **0.3** | 0.69 | 0.68 | 0.70 | 0.70 | 0.70 |
| **0.4** | 0.67 | 0.66 | 0.68 | 0.65 | 0.67 |
| **0.5** | 0.66 | 0.64 | 0.68 | 0.65 | 0.68 |

**Supplementary Table S5.** Parameter configuration of all datasets

| **Parameter in code** | **DLPFC** | **HBC** | **Slide-seqV2** | **Stereo-seq** | **MERFISH** |
| --- | --- | --- | --- | --- | --- |
| **epochs** | 400 | 200 | 400 | 350 | 250 |
| **lr** | 1e-3 | 1e-3 | 1e-3 | 1e-3 | 1e-3 |
| **weight_decay** | 1e-4 | 1e-4 | 1e-4 | 1e-4 | 1e-4 |
| **hidden_dim** | 512 | 512 | 512 | 512 | 512 |
| **output_dim** | 64 | 64 | 64 | 64 | 64 |
| ***α*** | 1e-2 | 1e-2 | 1.5e-3 | 1e-3 | 4e-4 |
| ***β₁*** | 1 | 1 | 1 | 1 | 1 |
| ***β_2_*** | 1 | 1 | 1 | 1 | 1 |
| **der** | 0.5 | 0.5 | 0.5 | 0.5 | 0.5 |
| **dfr** | 0.3 | 0.3 | 0.3 | 0.3 | 0.3 |

**Supplementary Table S6.** Performance of various loss terms on DLPFC

| **(α, *β₁, β_2_*)** | **ARI Mean** | **151673** | **151674** | **151675** | **151676** |
| --- | --- | --- | --- | --- | --- |
| **(1e-2, 1, 1)** | **0.63** | **0.66** | **0.63** | **0.57** | **0.64** |
| **(1e-2, 0.1, 1)** | 0.68 | 0.58 | 0.60 | 0.58 | 0.57 |
| **(1e-2, 0.1, 10)** | 0.57 | 0.56 | 0.58 | 0.57 | 0.57 |
| **(1e-2, 1, 0.1)** | 0.54 | 0.51 | 0.56 | 0.54 | 0.53 |
| **(1e-2, 1, 10)** | 0.42 | 0.42 | 0.43 | 0.43 | 0.39 |
| **(1e-2, 10, 0.1)** | 0.36 | 0.35 | 0.36 | 0.34 | 0.38 |
| **(1e-2, 10, 10)** | 0.27 | 0.25 | 0.27 | 0.27 | 0.28 |

**Supplementary Table S7.** Performance of various loss terms on DLPFC

| **(α, *β₁,β_2_*)** | **ARI Mean** | **151673** | **151674** | **151675** | **151676** |
| --- | --- | --- | --- | --- | --- |
| **(0, 1,1)** | 0.24 | 0.26 | 0.25 | 0.21 | 0.23 |
| **(1e-2, 0, 1)** | 0.28 | 0.28 | 0.24 | 0.30 | 0.29 |
| **(1e-2, 1, 0)** | 0.47 | 0.47 | 0.44 | 0.47 | 0.50 |

**Supplementary Table S8.** Performance of various HVGs on DLPFC

| **HVGs** | **ARI Mean** | **151673** | **151674** | **151675** | **151676** |
| --- | --- | --- | --- | --- | --- |
| **1000** | 0.36 | 0.37 | 0.37 | 0.36 | 0.35 |
| **2000** | 0.37 | 0.35 | 0.38 | 0.37 | 0.38 |
| **3000** | 0.41 | 0.39 | 0.43 | 0.39 | 0.42 |
| **4000** | 0.37 | 0.34 | 0.38 | 0.37 | 0.38 |
| **5000** | 0.40 | 0.38 | 0.40 | 0.41 | 0.42 |
| **6000** | 0.42 | 0.39 | 0.43 | 0.41 | 0.44 |
| **7000** | 0.54 | 0.51 | 0.56 | 0.54 | 0.53 |
| **8000** | 0.42 | 0.42 | 0.43 | 0.43 | 0.39 |
| **9000** | **0.63** | **0.66** | **0.63** | **0.57** | **0.64** |
| **10000** | 0.58 | 0.58 | 0.61 | 0.55 | 0.57 |

**Supplementary Table S9.** Performance of various HVGs on HBC

| **HVGs** | **ARI** | **NMI** | **HVGs** | **ARI** | **NMI** |
| --- | --- | --- | --- | --- | --- |
| **1000** | 0.59 | 0.71 | **6000** | 0.62 | 0.72 |
| **2000** | 0.57 | 0.71 | **7000** | 0.62 | 0.72 |
| **3000** | **0.64** | **0.73** | **8000** | 0.62 | **0.73** |
| **4000** | 0.63 | **0.73** | **9000** | 0.61 | 0.72 |
| **5000** | 0.63 | **0.73** | **10000** | 0.53 | 0.66 |

**Supplementary Table S10.** Performance of feature selection methods on DLPFC

| **HVGs Method** | **ARI Mean** | **151673** | **151674** | **151675** | **151676** |
| --- | --- | --- | --- | --- | --- |
| **Seurat V3** | **0.63** | **0.66** | **0.63** | **0.57** | **0.64** |
| **Seurat CCA** | 0.54 | 0.53 | 0.56 | 0.53 | 0.55 |

**Supplementary Table S11.** Number of neighbors per point in different datasets

| **Dataset** | **Mean neighbors** | **Dataset** | **Mean neighbors** | **Dataset** | **Mean neighbors** |
| --- | --- | --- | --- | --- | --- |
| **DLPFC**  **151507-151510** | 5.94 | **Merfish Sample2** | 5.98 | **Mob**  **Stereo-seq** | 5.96 |
| **DLPFC**  **151669-151672** | 5.97 | **Merfish Sample3** | 5.97 | **Mob**  **Slide-seqV2** | 5.97 |
| **DLPFC**  **151673-151676** | 5.98 | **Merfish Sample6** | 5.98 | **HBC** | 5.90 |

**Supplementary Table S12.** Comparison of training time and GPU memory usage across different methods on the DLPFC dataset

| **Method** | **Training time** | **GPU memory** |
| --- | --- | --- |
| **STGAT** | 3.08min | 6.26G |
| **STAligner** | 1.35min | 6.89G |
| **SEDR** | **10s** | **4.50G** |
| **STAGATE** | 3.48min | 10.48G |

**Supplementary Table S13.** Performance of methods on HBC

| **Method** | **ARI** | **NMI** |
| --- | --- | --- |
| **STGAT** | **0.64** | **0.73** |
| **stHGC** | 0.57 | 0.67 |
| **MVST** | 0.61 | 0.70 |
| **MMSpa** | 0.59 | 0.70 |
| **TOGAR** | 0.56 | 0.63 |
| **ST-GCP** | 0.57 | 0.64 |
| **Seurat** | 0.44 | 0.61 |
| **BayesSpace** | 0.49 | 0.68 |

**Supplementary Table S14.** Performance of various Different methods for constructing adjacency matrices on DLPFC

| **Method** | **ARI Mean** | **151673** | **151674** | **151675** | **151676** |
| --- | --- | --- | --- | --- | --- |
| **Delaunay triangulation** | **0.63** | **0.66** | **0.63** | **0.57** | **0.64** |
| **KNN (K = 5)** | 0.55 | 0.54 | 0.56 | 0.56 | 0.53 |
| **KNN (K = 6)** | 0.35 | 0.35 | 0.36 | 0.31 | 0.36 |
| **KNN (K = 7)** | 0.37 | 0.37 | 0.37 | 0.34 | 0.40 |
| **KNN (K = 8)** | 0.40 | 0.38 | 0.40 | 0.41 | 0.42 |
| **KNN (K = 9)** | 0.36 | 0.34 | 0.36 | 0.34 | 0.38 |
| **KNN (K = 10)** | 0.54 | 0.37 | 0.40 | 0.39 | 0.40 |
| **Radius (R = 3)** | 0.13 | 0.13 | 0.14 | 0.13 | 0.12 |
| **Radius (R = 4)** | 0.36 | 0.37 | 0.35 | 0.35 | 0.37 |
| **Radius (R = 5)** | 0.42 | 0.34 | 0.32 | 0.29 | 0.30 |
| **Radius (R = 6)** | 0.40 | 0.42 | 0.40 | 0.36 | 0.40 |
| **Radius (R = 7)** | 0.58 | 0.57 | 0.62 | 0.53 | 0.60 |
| **Radius (R = 8)** | 0.31 | 0.33 | 0.29 | 0.30 | 0.33 |
